# AID/APOBEC deaminases and cancer

**DOI:** 10.18632/oncoscience.155

**Published:** 2015-04-28

**Authors:** Stefan Rebhandl, Michael Huemer, Richard Greil, Roland Geisberger

**Affiliations:** ^1^ Department of internal Medicine III with Hematology, Medical Oncology, Hemostaseology, Infectious Diseases, Rheumatology, Oncologic Center, Laboratory for Immunological and Molecular Cancer Research, Paracelsus Medical University Salzburg, Austria; ^2^ Salzburg Cancer Research Institute, Salzburg, Austria

**Keywords:** deamination, cancer, AID, APOBEC3, mutation signature, mutation cluster, kataegis

## Abstract

Mutations are the basis for evolution and the development of genetic diseases. Especially in cancer, somatic mutations in oncogenes and tumor suppressor genes alongside the occurrence of passenger mutations have been observed by recent deep-sequencing approaches. While mutations have long been considered random events induced by DNA-replication errors or by DNA damaging agents, genome sequencing led to the discovery of non-random mutation signatures in many human cancer. Common non-random mutations comprise DNA strand-biased mutation showers and mutations restricted to certain DNA motifs, which recently have become attributed to the activity of the AID/APOBEC family of DNA deaminases. Hence, APOBEC enzymes, which have evolved as key players in natural and adaptive immunity, have been proposed to contribute to cancer development and clonal evolution of cancer by inducing collateral genomic damage due to their DNA deaminating activity. This review focuses on how mutagenic events through AID/APOBEC deaminases may contribute to cancer development.

## INTRODUCTION

Genomic DNA, composed of the four deoxynucleotides dA, dC, dG and dT, is being damaged constantly and therefore requires continuous DNA repair [1,2]. In particular, cytosine and 5-methylcytosine are susceptible to spontaneous hydrolytic deamination generating dU:dG and dT:dG mismatches leading to somatic and germline mutations, if not or improperly repaired [2,3]. Aside from spontaneous cytosine hydrolysis, uracils can also be introduced into DNA by polymerase-catalyzed mis-incorporation of dUTP instead of dTTP or through cytidine deaminase-mediated deamination of dC residues present in the DNA [4]. In higher eukaryotes, the dC deaminases all belong to the AID/APOBEC family of deaminases. In this review, the role of aberrant AID/APOBEC activity for cancer development is described.

### The AID/APOBEC family of deaminases

The AID/APOBEC family of proteins represents a group of cytidine deaminases that fulfills diverse physiological functions [5,6]. The protein family comprises eleven members in humans: AID and APOBEC1 (genes located on chromosome 12), APOBEC2 (gene located on chromosome 6), seven APOBEC3 proteins (APOBEC3A, APOBEC3B, APOBEC3C, APOBEC3D, APOBEC3F, APOBEC3G, APOBEC3H; genes located on chromosome 22) and APOBEC4 (gene located on chromosome 1) [7-12]. AID and APOBEC2 are considered the ancestral members of the AID/APOBEC family [5], whereas APOBEC1 and APOBEC3 appeared later as AID derivatives [5]. APOBEC3 proteins are detectable exclusively in mammals [5,13]. The gene copy number is species-specific, mice have a single APOBEC3 gene, while pigs (2), sheep (3), cattle (3), cats (4), horses (6) and primates (at least 7 APOBEC3 genes) have multiple copy numbers [5,10,13,14]. All members of the AID/APOBEC family share at least one zinc-binding catalytic domain with the consensus amino acid sequence H-X-E-X_23–28_-P-C-X_2–4_-C (X stands for any amino acid) [10,14,15].

The first member to be identified and characterized was APOBEC1, which deaminates mRNA for apolipoprotein B at cytosine^6666^ to uracil [16-18]. The resulting premature stop codon enerates apoB-48, which mediates uptake of dietary lipid from the intestine [16-18]. Additional mRNA targets for APOBEC1 have been described recently, where the interaction occurs at AU-rich sequence elements in 3′ UTRs and regulates mRNA stability [19,20]. Beside its role as an RNA editing enzyme [16], APOBEC1 is also capable of editing DNA in bacterial assays [21].

APOBEC2 was the second AID/APOBEC family member to be identified [9]. It is expressed in skeletal muscle and heart [9], but does not exhibit mutagenic activity in yeast and bacterial assays [22]. Although its precise physiological role has not been identified yet, APOBEC2 expression appears to be crucial for muscle development [23].

The group of APOBEC3 enzymes was first characterized by Jarmuz and co-workers as paralogs of APOBEC1 [10] and comprises seven proteins in humans (APOBEC3A-D, APOBEC3F, APOBEC3G, APOBEC3H) [12]. According to the number of the zinc-finger domains, APOBEC3 proteins can be classified in two groups: APOBEC3A, APOBEC3C and APOBEC3H have a single zinc-finger domain, whereas APOBEC3B, APOBEC3D, APOBEC3G and APOBEC3F harbor two zinc-finger domains [14,15]. APOBEC3 enzymes play an important role in innate immunity by inhibiting retroviruses through deamination of retroviral DNA intermediates [6]. In 2002, APOBEC3G was identified as the responsible factor for the restriction of Vif-deficient HIV-1 [24]. The retroviral restriction by APOBEC3 is counteracted by the Vif gene in human and rodent immunodeficiency viruses, as the Vif protein leads to degradation of APOBEC3 protein [24]. APOBEC3G is packaged into the HIV virions in an infected cell and in further consequence deaminates cytosines in the nascent DNA strand during viral reverse transcription in the newly infected target cell [6,15]. The uracil containing first strand then functions as template for second strand synthesis and thereby results in dG>dA hypermutations, which affect the viability of the virus and its ability to integrate into the host genome [6,15]. The Vif protein triggers polyubiquitination and subsequent degradation of APOBEC3G before virion incorporation [6]. But also APOBEC3B and APOBEC3F have been shown to have antiretroviral activity, at least for HIV-1 [25-28]. Aside from HIV, APOBEC3 proteins are also involved in defense against other viruses like human T-cell lymphotropic virus, hepatitis B virus, hepatitis C virus (HCV), human papillomavirus (HPV) and human herpesviruses [15]. Furthermore, APOBEC3 enzymes can restrict the movement of non-LTR and LTR retrotransposable elements, including long interspersed nuclear elements (LINEs) and short interspersed nuclear elements (SINEs) [29]. APOBEC3 proteins display distinct subcellular localization: APOBEC3B localizes to the nucleus [30-32], APOBEC3D, APOBEC3F and APOBEC3G are present in the cytoplasm [30,32], while APOBEC3A, APOBEC3C and APOBEC3H localize to both, nucleus and cytoplasm [32,33]. APOBEC3 proteins also mediate the clearance of exogenous double-stranded DNA (dsDNA) in human cells, as in a recent study APOBEC3A deaminated up to 97% of cytosines in foreign DNA leading to degradation, whereas genomic DNA remained unaffected [34]. The preferred sequence context for APOBEC3-mediated deamination is the TCW motif (W = A or T), in contrast to the APOBEC3G hot spot motif CC and the AID hot spot motif WRCY (R = A or G, Y = C or T) [35-41]. AID/APOBEC deaminases need single-stranded DNA (ssDNA) as substrate [6,41-47]. Quantitative profiling of APOBEC3 mRNA in 20 different human tissues, human T-cell lines and bulk leukocytes as well as leukocyte subsets demonstrated that APOBEC3 genes are expressed broadly and constitutively and that expression is not limited to immune cells [48]. However, APOBEC2^−/−^ and APOBEC3^−/−^ knockout mice display no obvious phenotype, suggesting that these genes are inessential for mouse development, viability or fertility [49]. Interestingly, recent work from Halemano and co-workers showed evidence that mouse APOBEC3 mutates immunoglobulin heavy chain variable genes (IgV) during retroviral infections, demonstrating thereby a previously unidentified function of APOBEC3 in generating virus-specific neutralizing antibodies and highlighting a new mechanism for antibody diversification in vivo [50]. In addition, APOBEC3 has also been attributed a role in double-strand break (DSB) DNA repair by non-homologous end joining (NHEJ) [51,52]. Expression of APOBEC3G in lymphoma cells associates with efficient DSB repair, whereas inhibition of APOBEC3G expression or deamination activity results in impaired joining of DSBs by NHEJ, implying a prosurvival role of APOBEC3 in lymphoma cells [51].

APOBEC4, the newest AID/APOBEC family member, is primarily expressed in testicles [11]. Its function is still unknown. Bacterial and yeast assays did not reveal mutagenic activity [22].

The ssDNA deaminating enzyme AID, which is primarily expressed in germinal center (GC) B cells, is the key deaminating enzyme for antigen-dependent antibody diversification through somatic hypermutation (SHM) and class switch recombination (CSR) [53-55]. There is a distinct evolutionary link between the vertebrate immune system and AID. This molecule is found in bony fish and cartilaginous fish, which are the first life forms to have an adaptive immune system [56]. AID deaminates dC thereby generating dU within the immunoglobulin (Ig) locus [53,55,57,58]. Since uracil base pairs with adenine, unrepaired uracils on the one hand lead to dC>dT transition mutations after DNA replication, whereas error prone DNA repair of uracil lesions on the other hand results in mutations at dC:dG as well as dA:dT base pairs [57]. The repair of deaminated cytosines within switch regions, 5′ of the constant regions of antibody genes, leads to the generation DSBs, which are a prerequisite for class switch recombination [59]. Hence, deficiency of functional AID in humans due to homozygous deletions or mutations causes the autosomal recessive form of an immunodeficiency syndrome, the hyper-IgM syndrome [53]. Disruption of AID in the chicken B cell lymphoma cell line DT40 abolishes antibody diversification by gene conversion [60]. First studies suggested that the enzyme edits RNA intermediates [61], before it could be shown in vitro that AID deaminates ssDNA during transcription [45,46,62], preferentially the dC within WRCY/RGYW motifs [41,63].

Aside from mutational activity, cytidine deaminases have also been shown to participate in epigenetics through active DNA demethylation [64]. Cytosines in DNA can occur either in an unmethylated or methylated (5-methylcytosine) form and are often enriched in promoter regions in a CpG dinucleotide context where methylation is associated with gene silencing [64]. Deamination of methylated cytidines generates thymidines, which are removed by the DNA repair machinery [64]. Insertion of a non-methylated dC would hence result in dC demethylation [64]. While recent studies suggest that AID is involved in promoter demethylation during early embryonic development [65-67], B cells from AID deficient mice did not exhibit any differences in methylated promoter regions [68].

### Regulation of AID activity

While APOBEC proteins execute deamination without cofactors and are regulated primarily on the level of gene expression, AID is the only family member which is tightly regulated on several layers, apparently because AID is the only deaminase which targets genomic DNA as physiological substrate and hence, off-target DNA damage is likely to occur. These layers of regulation are I) transcription and alternative splicing, II) posttranslational modification, III) shuttling, and IV) interaction partners that specify targeting to certain DNA loci.

Basically, transcription of AID is induced in B cells in the context of the GC reaction after helper T cell-mediated stimulation of B cells with CD40L and IL4 or TGFß [55], inducing the transcription factors NFĸB, Inhibitor of differentiation (Id) 2, Id3, the E-protein E47, PAX5, E2A and STAT6 [69-71]. Transcript levels are further regulated by alternative splicing, with only the full length AID variant being capable of mediating SHM and CSR [72-74]. As alternatively spliced AID transcripts are hardly detectable on protein level, alternative splicing is probably a further means to regulate the abundance of full length AID [75].

Concerning post translational modifications of AID, mass spectrometric and immunochemical approaches led to the identification of phosphorylation of several serine, threonine and tyrosine residues within the AID protein [76]. Of particular importance are amino acid residues S3 [77], S38 [76,78] and T140 [79], as their mutation to alanine does not impact catalytic activity, but interferes with CSR and SHM in vivo. This effect is particularly pronounced in AID haploinsufficient mice where AID levels are limited [80]. While phosphorylation of S38 was shown to be important for both processes, phosphorylation of T140 preferentially is necessary for somatic hypermutation of V region genes. In contrast, phosphorylation of S3 decreases AID activity [77]. In addition to phosphorylation, ubiquitination of AID was reported to occur thereby regulating protein abundance and activity [81,82]. AID continuously shuttles between cytoplasm and nucleus [83]. In particular, nuclear AID is efficiently polyubiquitinated followed by proteasomal degradation, while AID is relatively stable in the cytoplasm due to interaction with heat shock proteins [81,84-86]. Binding of nuclear AID to Reg-γ also induces an ubiquitin-independent degradative process [87]. Hence, nuclear cytoplasmic shuttling is a further level of regulating AID activity and its access to DNA substrates. Several proteins have been shown to interact with AID in B cells. Replication protein A (RPA), which is associated with transcription bubbles and binds and stabilizes ssDNA, interacts with phosphorylated AID and most likely helps AID recruitment to transcribed Ig regions [88]. AID also associates with UNG and MSH2/6 heterodimers, which is important for initiation and resolution of DSBs at switch regions [89]. The protein kinase A (PKA) alpha regulatory subunit (PKAr1α) was found to be responsible for phosphorylation of S38 of AID on a consensus PKA site, allowing interaction with RPA and representing an event critical for CSR [76]. The ubiquitin ligase MDM2 interacts with the C terminus of AID but most likely does not significantly regulate AID activity in vivo [90]. CTNNBL1, a spliceosomal protein, is a further interaction partner of AID [91]. Although CTNNBL1 was shown to substantially regulate SHM in DT40 cells, it did not affect CSR in a mouse B cell line [92]. A protein known to regulate splicing, PTBP2, was identified to promote binding of AID to the transcribed switch region and significantly reduces class switch efficiency under knockdown conditions [93]. AID is also associated with stalled RNA polymerase II, and their binding is dependent on SPT5, just as the recruitment of AID to the switch region, as shown by quantitative PCR-based ChIP analysis [94]. Moreover, SPT5 colocalizes with AID and its occupancy is predictive for AID-induced mutations [94]. The switch region contains repetitive 5‘-AGCT-3‘ motifs that are bound by the 14-3-3 adapter protein, which is upregulated in B cells undergoing CSR [95]. Blocking of 14-3-3 impedes AID binding to the switch region and results in decreased CSR [95]. Another protein specifically expressed in GC B cells is GANP. It plays an important role in localizing AID in the nucleus and promotes binding of AID to the transcribed IgV region and therefore supports AID in SHM [96].

Targeting of AID is further regulated by cis-acting elements. Aside from transcription of target genes as prerequisite for AID to gain access to ssDNA within the transcription bubble [45], recent evidence reveals convergent sense and antisense transcription within topologically complexed DNA cluster to recruit AID mediated DNA deamination [97-99].

### AID and cancer

A hallmark of B cell lymphomas is the occurrence of translocations between an oncogene and the Ig locus [100]. The most prominent example is the c-myc/IgH translocation in Burkitt's lymphoma. By sequencing this particular translocation junction, it has long been known that c-myc is directly joined to the switch region and hence, a role for aberrant CSR in mediating this translocation was hypothesized [101]. Soon after AID has been discovered as key enzyme for SHM and CSR, researchers aimed at determining its contribution to translocations as a result of “mistargeted CSR”. Indeed, subsequent studies revealed that c-myc/IgH translocations in IL6 transgenic mice are completely dependent on AID [102]. Moreover, by applying a deep-sequencing based method to document genome-wide chromosomal rearrangements in AID pro- and deficient mouse B cells, it became apparent that AID is responsible for translocations at multiple target genes, which are also found in human mature B cell lymphomas [103].

In addition to translocations, GC-derived lymphomas have been found to harbor many mutations in many non-Ig genes such as BCL6, CD79, CD95 and PAX5, which exhibit typical features of SHM [104-108]. Concomitantly, by comparing mutation frequencies in AID pro- versus deficient mice, it soon became clear that AID not only initiates the hypermutation of Ig genes but to a lesser extent also of many non-Ig genes, which are not properly protected by high-fidelity DNA repair, demonstrating off-target DNA damage induced by AID [109]. In addition, several reports show that many B cell lymphomas harbor subclonal heterogeneity at the Ig locus, which indicates ongoing AID activity during disease progression [110-113]. Hence, AID may not only contribute to malignant transformation but also to clonal evolution of B cell malignancies.

Aside from cancer of the B cell lineage, deregulated AID activity, mainly through overexpression, may induce various other types of cancer, such as gastric cancer [114,115], liver cancer [116], breast and ovarian cancer [117], lung cancer and T cell lymphomas [118]. A study from Morisawa and co-workers on organ-specific genetic profiles from different tumors (liver, stomach, lung) in AID transgenic mice demonstrated that AID-mediated genetic changes were organ-specific and may thereby contribute to tissue-specific genetic diversity during cancer development [119]. An increased rate of TP53 mutations was found to be associated with an overexpression of AID mRNA in human lung cancer cell lines [120]. A recent study from Shimizu and colleagues on human and murine gastric tumors and tissues shows that somatic mutations are enriched in multiple genes in gastric mucosal tissues upon *Helicobacter pylori* infection and that increased AID activity in these tissues leads to the accumulation of these mutations, which may promote carcinogenesis in *Helicobacter pylori* infected patients [121]. Dysregulated AID expression can also be induced by inflammation and microbial infections, which then acts as a genotoxic factor in various human cancers [122]. Inflammatory signals that induce epithelial to mesenchymal transition are capable of inducing AID expression [123]. Recent work presents evidence that this inflammation-induced AID expression is required for epithelial to mesenchymal transition in ZR75.1 breast cancer cells and non-transformed mammary epithelial cells, since AID deficiency in these cells suppresses expression of key transcriptional regulators for epithelial to mesenchymal transition and is associated with increased cytosine methylation in promoters of these genes [123]. This study proposes, in accordance with other reports [64,65,67], a role for AID in gene expression regulation and epigenetic reprogramming [123]. Furthermore, several oncogenic viruses can induce AID expression [124]. HCV, which is known to be a major cause of hepatocellular carcinoma, triggers AID expression in hepatocytes [116].

### APOBEC3 and cancer

A study performed by Alexandrov et al. showed the first evidence that aside from AID, also other APOBEC family members could contribute to genomic DNA damage [38]. Whole exome sequencing (WES) and whole genome sequencing (WGS) approaches of more than seven thousand human cancers revealed the presence of an APOBEC mutation signature, i.e. dC>dT transitions within a TCW (for APOBEC3) motif, in multiple cancer types, including bladder, breast, cervix and thyroid cancer, lung adenocarcinoma (LUAD), B cell lymphomas, multiple myelomas, acute lymphoblastic leukemia and chronic lymphocytic leukemia (CLL), whereas other cancers, like acute myeloid leukemia, liver and colorectal cancer, were devoid of such APOBEC signatures [38].

Moreover, a recent study from Burns and co-workers demonstrated that APOBEC3B mRNA is overexpressed in most primary breast tumors and breast cancer cell lines analyzed and that expression and activity of APOBEC3B correlated with genomic uracil levels, dC>dT transition rates and mutation frequencies [37]. They confirmed this correlation through knockdown of endogenous APOBEC3B, which is predominantly localized in the nucleus and showed that the induction of APOBEC3B overexpression in vitro results in extensive DNA damage, cell cycle arrest and finally cell death [37]. Also copy number variations affecting the APOBEC3 gene cluster on chromosome 22 might be important in breast cancer, since a germline copy number polymorphism, in which the gene-coding region of APOBEC3B is deleted and its 3′ UTR joined to APOBEC3A, associates with elevated risk of breast cancer [125,126]. This APOBEC3A-APOBEC3B germline polymorphism displays an elevated burden of APOBEC signature mutations, suggesting that this polymorphism presents cancer susceptibility through increased APOBEC activity [125]. Furthermore, APOBEC3B copy number alterations have been shown to be associated with decreased APOBEC3B expression in breast cancer cell lines [127]. When introducing APOBEC3A and APOBEC3B into yeast, genome-wide mutation patterns of APOBEC3A- and APOBEC3B-mediated deamination show strong similarity to mutation signatures found in breast cancer, which strengthens the proposed role of APOBEC3B, and maybe also APOBEC3A, in breast cancer hypermutation [35]. APOBEC3B was shown to be upregulated in several human lymphoma cell lines where cells with high APOBEC3B expression possessed mutations, mainly dC>dT transitions, in actively transcribed oncogenes [128]. Also in CLL, APOBEC3B mutation signatures have been identified [129]. Overexpression of APOBEC3B in a APOBEC3B low expressing lymphoma cell line induced accumulation of dC>dT mutation within the c-myc gene [128]. APOBEC3B is furthermore elevated in lung cancer, ovarian carcinoma, diverse ovarian cancer cell lines and high grade primary ovarian cancers [130,131]. WGS and expression profiling of 16 primary ovarian tumors revealed a correlation between APOBEC3B mRNA expression and total mutation load as well as transversion levels [130]. Due to the expression data and mutation patterns, it was suggested that APOBEC3B lesions are processed error prone and also attribute APOBEC3B a potential role in genomic instability in serous ovarian cancer [130]. It is also likely, that APOBEC3 contributes to tumor progression in lung cancer, as a recent study demonstrated that a substantial amount of subclonal driver mutations occurred in an APOBEC sequence context [132]. Interestingly, increased APOBEC-associated mutation levels were found specifically in the region of LUAD, where the highest APOBEC3B mRNA levels were detected [132]. A cooperative role in APOBEC3B mutagenesis is furthermore attributed to the tumor suppressor fragile histidine triad protein (FHIT), which is known to cause DNA damage upon loss of protein activity in normal and cancer cells [133]. Frequencies of APOBEC3-mediated mutations were shown to vary upon changes in FHIT expression [133]. LUADs with low FHIT and high APOBEC3B expression harbored an increased number of APOBEC signature mutations, whereas samples with normal FHIT and high APOBEC3B expression did not show hypermutation due to APOBEC activity [133]. It was suggested that different levels of APOBEC3B and FHIT expression result in increased or decreased frequencies of APOBEC-mediated mutations [133].

Very recently, APOBEC3 proteins have been suggested to link viral infections to cancer development [134]. Stable transfection of normal breast epithelial cells with HPV was demonstrated to cause APOBEC3B mRNA overexpression leading to a significant increase in γ-H2AX foci formation and DNA breaks, which were blocked by knockdown of HPV and APOBEC3B [135]. For head and neck squamous cell carcinomas (HNSCCs) it has been shown by Henderson and colleagues that HPV^+^ compared to HPV^−^ tumors have elevated APOBEC3B and that PIK3CA helical domain mutations are APOBEC-driven in multiple cancers, including HPV^+^ HNSCC [136]. In addition, the authors describe a role for APOBEC-mediated mutagenesis in HPV-driven tumor development [136].

But also APOBEC3 proteins aside from APOBEC3B are reported to be implicated in cancer. APOBEC3G was found to be highly expressed in colorectal tumors and hepatic metastasis and proposed to promote colorectal cancer hepatic metastasis through miR29 downregulation and consequent derepression of MMP2, a known metastasis activator [137]. Additionally, APOBEC3G acts as a prosurvival factor in lymphoma cells [51,52]. An inhibition of APOBEC3G expression results in DSB repair deficiencies, which can be reconstituted by elevated APOBEC3G levels, suggesting that APOBEC3G recognizes dsDNA ends and facilitates rejoining through NHEJ [51,52]. In a gene expression study comprising 67 diffuse large B cell lymphoma patients treated with R-CHOP (rituximab-cyclophosphamide, doxorubicin, vincristine, prednisone), a set of 16 genes, including APOBEC3G, was identified to correlate with shortened overall survival and progression free survival [138]. Recent data link APOBEC3A activity to skin lesions, where APOBEC3-mediated transcription-dependent mutations, apart from CC dimer formation through UV radiation, are considered alternative initiating events in skin cancer [139]. Summarizing, APOBEC3 proteins are key players in cancer-associated somatic mutation processes and seem to influence cancer development and progression in several entities.

### Mutation clusters (‘kataegis’) in cancer

An analysis of 21 breast cancer genomes uncovered the phenomenon of localized hypermutation, termed ‘kataegis’ (Greek for ‘shower’ or ‘thunderstorm’), with mostly dC>dT transitions at TpC dinucleotides, suggesting APOBEC3 proteins to be implicated in the formation of these mutation clusters [36]. Accumulated mutations have already been observed before, when Wang and colleagues sequenced the LacZ gene in spontaneous derived tumors from the Big Blue mouse and found multiple mutations with short intermutational distances [140]. Kataegis in many cancer types are associated with APOBEC3 enzymes, since mutation patterns are dominated by dC>dT transitions and dC>dG transversions, that occur within preferred APOBEC3 motifs [38]. APOBEC3B and APOBEC3A expression levels have been shown to correlate with kataegic mutation burden [141,142]. In vitro induction of mutation showers in yeast was observed upon expression of PmCDA1, a highly mutagenic cytosine deaminase from sea lamprey [143], or AID and APOBEC3B [35]. Although the deficiency of UNG dramatically increased the number of dC>dT transitions in these experiments, the number of dC>dG transversions as well as the amount of kataegic events was drastically reduced [35]. The same effect was observed in the absence of the error prone DNA polymerase REV1, which is able to insert nucleotides opposed to abasic sites with a strong preference for inserting dCs, thus resulting in dC>dG transversions [35].

**Figure 1:**
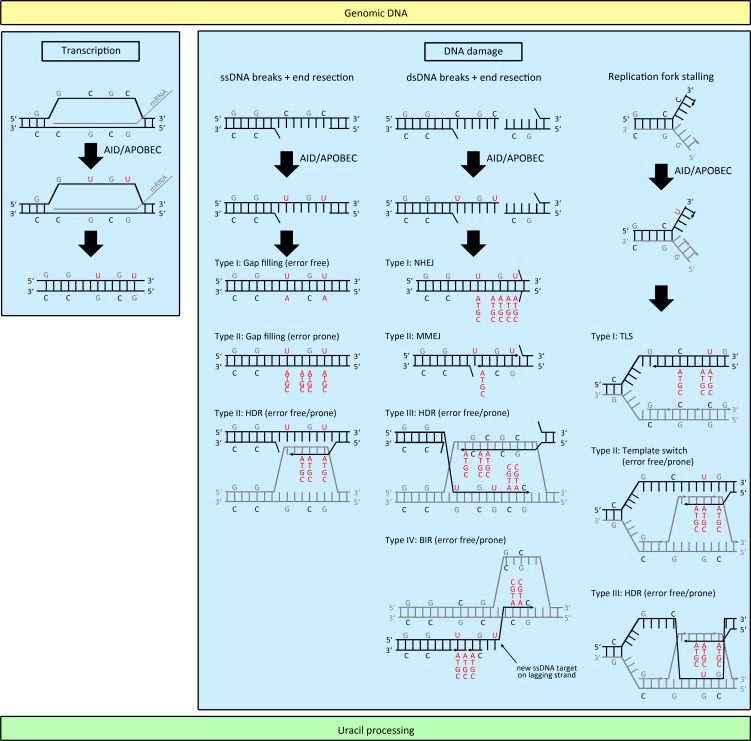
AID/APOBEC mediated mutagenesis of genomic DNA Efficient dC deamination requires generation of ssDNA within genomic DNA. ssDNA can be generated by either transcription, DNA damage, which eventually leads to SSB and DSB, or by DNA replication in which replication fork stalling due to DNA lesions extends ssDNA formation. In either case, dUs generated on ssDNA remain in the dsDNA during error free or error prone repair of the initial lesion, leading to dU/dN mismatches. (Abbreviations: SSB: single-strand break; DSB: double-strand break; HDR: homology-directed repair; NHEJ: non-homologous end joining; MMEJ: microhomology-mediated end joining; BIR: break-induced replication; TLS: translesion synthesis).

**Figure 2:**
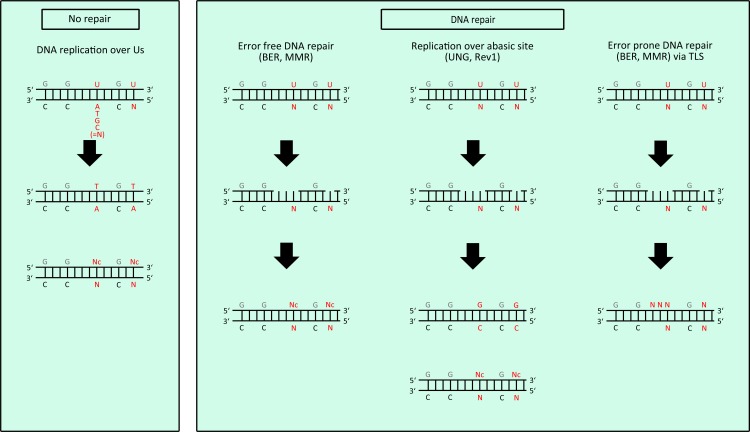
Processing of uracils within genomic DNA dU/dN mismatches generated as described in Figure 1 are processed by different mechanisms: Replication of dU/dN mismatches eventually leads to dT/dA pairing (left panel). DNA repair (BER, MMR) can either occur error free or error prone, depending on which DNA polymerases are recruited. In case dUs are removed by UNG, replication over an abasic site often leads to the REV1 based incorporation of dCs opposite to the abasic site, which results in dC>dG transversions. (Abbreviations: BER: base excision repair; MMR: mismatch repair; TLS: translesion synthesis; Nc: complementary base to N).

Hence, a functional DNA repair machinery is a prerequisite for the induction of kataegis. The prevailing explanation for this observation is that DNA repair generates ssDNA substrates for APOBEC proteins during ssDNA resection (Figure 1). This means that DNA lesions either initiated through APOBEC enzymes themselves or by any other damaging agents (UV-light, radiation, ROS, etc.) induces DNA repair by either BER or MMR, which comprise 5′-3′ resection of the damaged DNA strand, leading to the generation of long ssDNA stretches that are attacked by APOBEC proteins. This repair-induced mutagenesis thus leads to strand-coordinated mutations, whereas only dCs on the non-resected DNA strand are deaminated, and to clusters of mutations, where mutations are focused on regions of a few kilobases, which are typically resected during DNA repair [144,145]. Interestingly, a portion of mutation clusters colocalizes with breakpoints of chromosomal rearrangements, DSBs or uncapped telomere ends [35,36,146,147]. During DSB repair, dsDNA ends can either be directly ligated by NHEJ or ssDNA is formed by 5′-3′ end resection which facilitates homology-directed repair through microhomology-mediated end joining (MMEJ), homologous recombination or break-induced replication (BIR). In these cases, the generated ssDNA is again a substrate for APOBEC enzymes [35,36,146,147]. During BIR, a DSB is repaired by replication starting from the DSB using the homologous dsDNA strand as template [148,149]. As this repair pathway was reported to have a drastically delayed lagging strand compared to leading strand synthesis, ssDNA during lagging strand synthesis has a higher propensity to be attacked by APOBEC deaminases [148,149]. In this regard, any delay in lagging strand synthesis induced by e.g. polymerase stalling or interchain crosslink increases the likelihood that ssDNA within the replication fork is attacked by APOBEC proteins [148]. As a consequence of any attack by AID/APOBEC members, dUs are accumulating in ssDNA, which possibly remain in dsDNA even after DNA repair (Figure 1). Replication over these dUs eventually results in dC>dT mutations, whereas replication over abasic sites (in case dU is removed by UNG prior replication) is mainly accomplished by REV1, which preferentially incorporates dCs opposite to the abasic site, leading to dC>dG transversions (Figure 2). Both mutations are typically enriched within kataegic events. Alternatively, dUs can be repaired by BER or MMR, where dUs are removed during 5′-3′ resection of ssDNA batches followed by high fidelity gap filling by Polδ or otherwise, by error prone gap filling by translesion synthesis (TLS) polymerases. The recruitment of error prone TLS polymerases is the main mechanism that provides AID-induced SHM of antibody variable genes [57] (Figure 2).

According to the literature it can be stated that the AID/APOBEC family of deaminases is likely to play an important role in mutagenesis and clonal evolution in cancer and moreover to be a key factor for kataegic events in cancer genomes. A major future challenge will be to assess whether presence of AID/APOBEC enzymes is sufficient to support mutagenesis or whether additional factors facilitate deaminase recruitment and accumulation of mutations.
